# Post-diagnostic beta blocker use and breast cancer-specific mortality: a population-based cohort study

**DOI:** 10.1007/s10549-022-06528-0

**Published:** 2022-03-14

**Authors:** Oliver William Scott, Sandar Tin Tin, J. Mark Elwood, Alana Cavadino, Laurel A. Habel, Marion Kuper-Hommel, Ian Campbell, Ross Lawrenson

**Affiliations:** 1 Department of Epidemiology and Biostatistics, School of Population Health, University of Auckland, Building 507, 85 Park Road, Grafton, Auckland, 1023 New Zealand; 2 Division of Research, Kaiser Permanente Northern California, Oakland, CA USA; 3 Department of Oncology, Waikato District Health Board, Hamilton, New Zealand; 4 Department of Strategy, Investment and Transformation, Waikato District Health Board, Hamilton, New Zealand; 5 Breast and General Surgeon, Waikato Hospital, Hamilton, New Zealand; 6 Department of NIDEA (National Institute of Demographic and Economic Analysis), Waikato Medical Research Centre, University of Waikato, Hamilton, New Zealand

**Keywords:** Breast cancer, Mortality, Beta blockers, Pharmacoepidemiology, Cohort study

## Abstract

**Purpose:**

Beta blockers (BB) have been associated with improved, worsened, or unchanged breast cancer outcomes in previous studies. This study examines the association between the post-diagnostic use of BBs and death from breast cancer in a large, representative sample of New Zealand (NZ) women with breast cancer.

**Methods:**

Women diagnosed with a first primary breast cancer between 2007 and 2016 were identified from four population-based regional NZ breast cancer registries and linked to national pharmaceutical data, hospital discharges, and death records. The median follow-up time was 4.51 years. Cox proportional hazard models were used to estimate the hazard of breast cancer-specific death (BCD) associated with any post-diagnostic BB use.

**Results:**

Of the 14,976 women included in analyses, 21% used a BB after diagnosis. BB use (vs non-use) was associated with a small and nonstatistically significant increased risk of BCD (adjusted hazard ratio: 1.11; 95% CI 0.95–1.29). A statistically significant increased risk confined to short-term use (0–3 months) was seen (HR = 1.40; 1.14–1.73), and this risk steadily decreased with increasing duration of use and became a statistically significant protective effect at 3 + years of use (HR = 0.55; 0.34–0.88).

**Conclusion:**

Our findings suggest that any increased risk associated with BB use may be driven by risk in the initial few months of use. Long-term BB use may be associated with a reduction in BCD.

**Supplementary Information:**

The online version contains supplementary material available at 10.1007/s10549-022-06528-0.

## Introduction

Breast cancer is the most common cancer in women and the leading cause of female cancer mortality worldwide [1]. Comorbidities are common in patients with breast cancer [2], and there is a high and increasing prevalence of risk factors for both breast cancer and ischemic heart disease among Western women [3–5]. As such, many patients with breast cancer use prescribed medications for cardiovascular conditions. Examining the association between commonly used cardiovascular medications and breast cancer outcomes is therefore warranted. Beta blockers (BBs), principally indicated for angina, arrhythmias, heart failure, hypertension, and myocardial infarction [6, 7], are commonly used cardiovascular medications that have been used in Western medicine for decades [8–10].

Human breast cancer cells have beta-adrenergic receptors [11]. Responses induced by beta-adrenergic signalling include upregulated expression of metastasis-associated genes involved in inflammation, angiogenesis, and tissue invasion, and downregulated expression of genes facilitating anti-tumour immune responses [12]. Beta-adrenergic receptors mediate the catecholamine hormones produced in the stress response, which in turn produce the aforementioned responses [13]. These hormones can be blocked by BBs, resulting in a potential protective effect through interference with tumour cell proliferation and migration, as well as tumoural angiogenesis [14, 15]. On the basis of this evidence, several observational studies have been carried out, and some have reported that BB use may be protective for breast cancer-specific death (BCD) [16–19]. However, others have noted no association between BBs and BCD [20–25], and one reported BBs were associated with an increased risk of BCD [26]. The only RCT carried out on BBs (to date) found that preoperative propranolol downregulated biomarkers of invasive potential and inflammation, and improved biomarkers of cellular immune response [27].

There have been several methodological limitations in previous studies examining the association between BB use and BCD. For example, some studies fail to account for the time-varying nature of medication use [28]. Moreover, there have been few studies which have explored the dose–response effect of BBs. Therefore, our primary objective was to address these limitations and explore the relationship between any post-diagnostic BB use and BCD in a large population-based cohort study of newly diagnosed patients with breast cancer in New Zealand.

## Methods

### Data sources

Eligible women were all those with a first primary breast cancer diagnosed and recorded in any of four population-based regional breast cancer registries (Auckland, Waikato, Wellington, and Christchurch) [29] in New Zealand between 1 Jan 2007 and 31 Dec 2016. These registers include all women diagnosed with breast cancer in their defined areas and together cover about 70% of all breast cancer registrations in New Zealand. Using an anonymised National Health Index number, data were linked to several national data bases: the Pharmaceutical Collection (PHARMS), a national database containing dispensing information and medication identifiers from pharmacists for subsidised dispensings [30]; the National Minimum Dataset, relating to all day patients and inpatients discharged from both public and private hospitals; and the National Mortality Collection, with information about all certified deaths [31]. Women were excluded if their records did not link to at least one dispensing from the pharmaceutical collection (*n* = 14) or if their date of death was on or before their recorded date of breast cancer diagnosis (*n* = 3). The final cohort for analyses was composed of 14,976 women.

### Exposure and outcome data

In the PHARMS database, medications dispensed any time after breast cancer diagnosis were determined using the therapeutic group ID, a PHARMAC identifier for each group of Anatomical, Therapeutic, and Chemical properties [30]. All BBs were included (including both selective and non-selective), except those used topically for glaucoma. For each dispensing, we calculated the total dose dispensed in mg by multiplying the number of tablets dispensed by the dose per tablet, and then converted this to the number of ‘daily defined doses’ as defined by the World Health Organisation database [32].

Deaths were determined from the underlying cause of death in the regional breast cancer registries and National Mortality Collection, with ICD codes C50.0 to C50.9 classified as deaths from breast cancer.

### Confounders

Demographic and clinical information came from the regional breast cancer registries, and covariates considered included date of diagnosis, age, ethnic group [33, 34], socioeconomic deprivation (NZDep, a measure of socioeconomic deprivation based on small area census data, ranging from 1 (least deprived) to 10 (most deprived) [35]), urban/rural status [36], public/private status of the treatment facility, register, stage [37], grade [38], mode of detection (screen detected vs symptomatic), lymphovascular invasion, and receptor status (as defined previously [39], including Luminal A, Luminal B HER2-, Luminal B HER2 +, HER2 + non-luminal, and triple negative). Other post-diagnostic medications included statins, aspirin and other non-steroidal anti-inflammatory medications (NSAIDs), angiotensin-converting enzyme inhibitors (ACEIs), angiotensin receptor blockers (ARBs), and diuretics. Comorbidities adjusted for included any cardiac condition (angina, arrhythmia, congestive heart failure, hypertension, myocardial infarction, ‘other cardiac conditions’, and valve disease) as yes/no, diabetes, stroke, chronic obstructive pulmonary disorder, and peripheral vascular disease. We defined comorbidities as any of the above conditions appearing in a patient’s linked hospital record (inpatient admissions) in the 5-year period before their breast cancer diagnosis.

### Statistical analyses

Comparisons by BB use at baseline (date of diagnosis of breast cancer) were conducted using the chi-square test. We used Cox proportional hazard models to assess hazard ratios (HRs) of breast cancer-specific mortality associated with any post-diagnostic BB use vs non-use. Death registrations and Pharmaceutical Collection coverage were complete to the end of 2017, so we followed patients from their breast cancer diagnosis until death or 31 December 2017. Women with no death recorded prior to 31 December 2017 were assumed to be alive as at 31 December 2017. Medication use was conceptualised as a time-varying covariate, such that time before the first dispensing was counted as ‘nonuser’ time, and time from the first dispensing to end of followup was counted as ‘user’ time [40]. Models were adjusted in a systematic fashion, with the first adjustment including demographic and breast cancer clinical data, and the second adding other medication use and comorbidities.

Analyses were conducted considering BB use as a binary variable (user/nonuser), and also by splitting BB use into seven categories based on the number of daily defined doses (DDDs: categorised as 1–90 DDDs, 91–181 DDDs, 182–272 DDDs, 273–364 DDDs, 365–729 DDDs, 730–1094 DDDs, or 1095 or more DDDs, corresponding to the equivalent of 0–3 months, 3–6 months, 6–9 months, 9 months-1 year, 1–2 years, 2–3 years, and 3 + years of BB use, respectively). Dose analyses were conducted using a time-varying. Approach, such that women spent time in the lowest category before moving into the next dose category. In order to compare patterns of risk observed for BBs to those of another cardiovascular medication with similar indications [41], the same analyses were carried out for calcium channel blockers.

To examine the effect of BB use in early-stage patients only, an analysis was carried out restricted to patients with stage 1, stage 2, or stage 3a cancers. In this analysis, patients with an ‘unknown’ stage were excluded.

To evaluate the effect of the competing risk of death from other causes, the proportional subhazards model was also used [42]. For this analysis, all deaths apart from breast cancer deaths were treated as competing events.

As dispensings towards the end of life may reflect changes in morbidity (including cancer recurrence/progression) or in health care related to end-of-life care [43, 44], we also conducted analyses lagging medication times [45]. In these analyses, patients are initially considered nonusers and then users after a lag period has elapsed after their first medication dispensing. Using this approach, dispensings towards the end of life are removed by the lag; for example, a 6-month lag will ignore dispensings in the 6 months prior to death/last follow-up and classify these women as medication nonusers as opposed to users. To appropriately account for different periods in which end-of-life care may be administered, we also considered lag periods of 1 year and 2 years. In these analyses, all medications were modelled in the same fashion (for example, if BBs were lagged by 6 months, all other medications were as well).

In order to compare BB users to patients using other medications for a similar indication, a further analysis was carried out comparing BB users to BB nonusers who used another antihypertensive medication. For this comparison, other antihypertensives included ‘Potassium Sparing Combination Diuretics’, ‘Thiazide and Related Diuretics’, ‘ACE Inhibitors’, ‘ACE Inhibitors with Diuretics’, ‘Angiotensin II Antagonists’, ‘Angiotensin II Antagonists with Diuretics’, ‘Dihydropyridine Calcium Channel Blockers’, ‘Other Calcium Channel Blockers’, ‘Alpha Adrenoceptor Blockers’, and ‘Centrally Acting Agents’. In this analysis, BB nonusers who used another antihypertensive were followed from their first post-diagnostic antihypertensive dispensing until death or 31 December 2017.

We also conducted an analysis with breast cancer recurrence (BCR) as the outcome. In this analysis, we defined a BCR as either a local/regional recurrence or distant metastasis and restricted the cohort to patients with early-stage breast cancer as above. Recurrences were determined from the breast cancer registry data through patient’s routine clinical records, and women were followed from their breast cancer diagnosis until BCR, death, last follow-up date, or end of Pharmaceutical Collection coverage (31 December 2017), whichever came first. These analyses examined the risk of BCR associated with BB use vs non-use, as well as the risk associated with different doses of BB use vs non-use.

Results are reported as HRs and their 95% confidence intervals (CIs), with the two-sided significance level set at 0.05. Statistical analyses were conducted in STATA 13.1 (StataCorp, College Station, TX).

## Results

Median follow-up for our cohort of 14,976 women was 4.51 years (range 0.01–10.99 years), with 1341 dying of breast cancer, and 884 dying from other causes. Of these 14,976 women, 21% were dispensed a BB after diagnosis (Table 1). Higher proportions of BB users compared to nonusers were diagnosed in earlier years of the study period, were older, were from more deprived areas, and were treated in a public facility. A higher proportion of BB users than nonusers were also more likely to have used other medications (statins, aspirin, ACEIs, ARBs, and diuretics) and to have had documented comorbidities (any cardiac condition, diabetes, stroke, COPD, and peripheral vascular disease) (all statistically significant differences, *p* < 0.05).

**Table 1:** Characteristics of breast cancer patients by beta blocker use

Characteristics	Beta blocker use after diagnosis	
	Ever-*n* (%)	Never-*n* (%)
Overall	3195	11,781
Year of diagnosis		
2007–2008	534 (17)	1393 (12)
2009–2010	617 (19)	2039 (17)
2011–2012	723 (23)	2546 (22)
2013–2014	693 (22)	2823 (24)
2015–2016	628 (20)	2980 (25)
Age at diagnosis		
< 50	323 (10)	3933 (33)
50–59	609 (19)	3389 (29)
60–69	1038 (32)	2789 (24)
70–79	706 (22)	1072 (9)
80 +	519 (16)	598 (5)
Ethnic group		
European	2425 (76)	8603 (73)
Maori	325 (10)	1103 (9)
Pacific	187 (6)	761 (6)
Asian	182 (6)	1033 (9)
Other	66 (2)	281 (2)
NZDep^a^		
1–2	431 (13)	2208 (19)
3–4	576 (18)	2345 (20)
5–6	685 (21)	2319 (20)
7–8	513 (16)	1744 (15)
9–10	571 (18)	1643 (14)
Unknown	419 (13)	1522 (13)
Urban/rural		
Urban	2551 (80)	9352 (79)
Rural	227 (7)	914 (8)
Unknown	417 (13)	1515 (13)
Status of facility		
Public	2333 (73)	7594 (64)
Private	862 (27)	4187 (36)
Register		
Auckland	1675 (52)	6581 (56)
Christchurch	506 (16)	1918 (16)
Waikato	607 (19)	1638 (14)
Wellington	407 (13)	1644 (14)
Cancer stage		
1	1378 (43)	5368 (46)
2	1120 (35)	4004 (34)
3	397 (12)	1533 (13)
4	141 (4)	555 (5)
Unknown	159 (5)	321 (3)
Cancer grade		
Well differentiated	660 (21)	2613 (22)
Moderately differentiated	1496 (47)	5061 (43)
Poorly differentiated	859 (27)	3455 (29)
Unknown	180 (6)	652 (6)
Method of diagnosis		
Symptomatic	1953 (61)	6815 (58)
Screen detected	1242 (39)	4966 (42)
Lymphovascular invasion		
No	1881 (59)	7064 (60)
Yes	1050 (33)	4023 (34)
Unknown	264 (8)	694 (6)
Receptor status		
HER2+ non-luminal	142 (4)	639 (5)
Luminal A	2074 (65)	7872 (67)
Luminal B HER2-	116 (4)	274 (2)
Luminal B HER2+	246 (8)	1079 (9)
Triple negative	324 (10)	1219 (10)
Unknown	293 (9)	698 (6)
Other medication use after diagnosis		
Statins	1667 (52)	2393 (20)
NSAIDs	1951 (61)	8377 (71)
Aspirin	1744 (55)	2137 (18)
ACEIs	1718 (54)	2410 (20)
ARBs	612 (19)	741 (6)
Diuretics	1546 (48)	1879 (16)
Hospitalised comorbidities^b^		
Any cardiac condition	740 (23)	539 (5)
Diabetes	246 (8)	319 (3)
Stroke	133 (4)	127 (1)
COPD	70 (2)	138 (1)
Peripheral vascular disease	46 (1)	24 (0.2)

^a^The NZDep is an area-based measure of socioeconomic deprivation in New Zealand. 1 represents the areas with the least deprived scores and 10 the areas with the most deprived scores

^b^Comorbidities included those in a patient’s hospital records five years before breast cancer diagnosis. Cardiac conditions included any of angina, arrhythmia, congestive heart failure, hypertension, myocardial infarction, ‘other cardiac conditions’, and valve disease

The chi-square test was statistically significant (*p* < 0.05) for every variable except for urban/rural

We compared the risk of BCD associated with BB use (vs non-use) after diagnosis (Table 2). In the unadjusted model, BB use was associated with an increased risk of BCD (HR = 1.49; 95% CI 1.31–1.70). This increased risk was markedly reduced after adjustment for demographic and breast cancer clinical factors (HR = 1.16; 1.01–1.33), and modestly reduced with further adjustment for other medication use and comorbidities, such that there was only a small and nonstatistically significant increased risk in the fully adjusted model (HR = 1.11; 0.95–1.29). The fully adjusted HR was similar in the analysis restricted to patients with early-stage cancers only (HR = 1.17; 0.94–1.46). A similar fully adjusted finding was noted when adjusting for the competing risk of death from other causes (HR = 1.06; 0.88–1.26). Lagging BB use by various lengths of time did not substantially alter the HR (Table 2). When adjusting for demographic variables, clinical variables, comorbidities, and other medication use in four stages, it was found that demographic variables were the strongest confounders of the association (Supplementary Table S1).

**Table 2:** Associations of breast cancer-specific survival with post-diagnostic use of beta blockers (vs non-use) in breast cancer patients, by total dose

Medication usage after diagnosis	No. breast cancer deaths	No. person-years	Unadjusted HR (95% CI)	Adjusted^a^ HR (95% CI)	Fully adjusted^b^ HR (95% CI)
BB nonuser	1036	60,301	1.00	1.00	1.00
BB user	305	12,487	1.49 (1.31–1.70)	1.16 (1.01–1.33)	1.11 (0.95–1.29)
BB nonuser, early stage^c^	446	54,962	1.00	1.00	1.00
BB user, early stage^c^	151	11,223	1.58 (1.32–1.91)	1.29 (1.06–1.58)	1.17 (0.94–1.46)
BB user, adjusting for competing risks	305	12,487	1.43 (1.26–1.63)	1.11 (0.94–1.31)	1.06 (0.88–1.26)
BB user, 6-month lag	252	10,946	1.38 (1.20–1.59)	1.06 (0.92–1.24)	1.14 (0.97–1.35)
BB user, 1-year lag	220	9496	1.41 (1.21–1.63)	1.09 (0.93–1.28)	1.22 (1.03–1.46)
BB user, 2-year lag	131	6965	1.21 (1.00–1.46)	0.94 (0.77–1.14)	1.05 (0.84–1.31)
1–90 DDDs (0–3 months)	109	3201	2.09 (1.72–2.55)	1.54 (1.26–1.89)	1.40 (1.14–1.73)
91–181 DDDs (3–6 months)	45	1782	1.44 (1.07–1.94)	1.24 (0.91–1.68)	1.15 (0.84–1.57)
182–272 DDDs (6–9 months)	31	1225	1.42 (0.99–2.04)	1.26 (0.88–1.81)	1.22 (0.85–1.77)
273–364 DDDs (9 months–1 year)	27	1001	1.50 (1.02–2.20)	1.19 (0.81–1.75)	1.14 (0.77–1.68)
365–729 DDDs (1 year–2 years)	53	2325	1.33 (1.01–1.76)	1.01 (0.76–1.35)	0.96 (0.71–1.28)
730–1094 DDDs (2 years–3 years)	21	1180	1.12 (0.73–1.73)	0.77 (0.49–1.19)	0.78 (0.50–1.22)
1095 or more DDDs (3 or more years)	19	1774	0.81 (0.51–1.28)	0.54 (0.34–0.86)	0.55 (0.34–0.88)

^a^First adjustment controlled for date of dx, age, ethnic group, deprivation, urban/rural status, public/private status of the facility, register, stage, grade, mode of detection, lymphovascular invasion, and receptor status

^b^Second adjustment controlled for the previous covariates as well as other drug use and hospitalised comorbidities (other drugs including statins, NSAIDs and aspirin, ACEIs, ARBs, and diuretics. Comorbidities including any cardiac condition as yes/no, diabetes, stroke, COPD, and peripheral vascular disease). Other drug covariates were modelled in the same fashion as beta blockers (except for dose analysis, in which other drugs were classified as user/nonuser and modelled as time-varying covariates)

^c^Restricted to patients with stage 1, stage 2, or stage 3a cancers. Patients with an ‘unknown’ stage were excluded in this analysis

^d^DDDs refer to daily defined doses

^e^The p value for linear trend for the fully adjusted dose analysis was 0.0005

In the dose analysis, the highest risk was observed during the initial 0–3 months of BB use (fully adjusted HR = 1.40; 1.14–1.73, Table 2). The risk decreased but remained elevated for use up to a year, after which the risk was reduced with increasing duration of use, and a 45% reduction in BCD was found in those who took a BB for the equivalent of 3 or more years (HR = 0.55; 0.34–0.88). Excluding nonusers (i.e. among users only), the overall *p* value for linear trend was 0.0005. In the same analysis with calcium channel blockers as the exposure of interest (Table 3), use of less than 3 months was not associated with an elevated risk and risk did not consistently decrease with increasing duration of use among users (*p* for trend = 0.2).

**Table 3:** Associations of breast cancer-specific survival with post-diagnostic use of calcium channel blockers (vs non-use) in breast cancer patients, by total dose

Medication usage after diagnosis	No. breast cancer deaths	No. person-years	Unadjusted HR (95% CI)	Adjusted^a^ HR (95% CI)	Fully adjusted^b^ HR (95% CI)
CCB nonuser	1100	61,265	1.00	1.00	1.00
CCB user	241	11,523	1.23 (1.07–1.41)	0.94 (0.81–1.10)	0.89 (0.76–1.05)
1–90 DDDs (0–3 months)	40	1750	1.34 (0.98–1.84)	0.82 (0.59–1.14)	0.84 (0.60–1.16)
91–181 DDDs (3–6 months)	25	1121	1.32 (0.88–1.96)	0.98 (0.65–1.46)	0.88 (0.59–1.33)
182–272 DDDs (6–9 months)	30	882	1.89 (1.31–2.72)	1.52 (1.05–2.19)	1.48 (1.02–2.14)
273–364 DDDs (9 months–1 year)	23	837	1.50 (0.99–2.27)	1.26 (0.83–1.92)	1.18 (0.78–1.81)
365–729 DDDs (1 year–2 years)	44	2257	1.05 (0.77–1.42)	0.83 (0.61–1.12)	0.78 (0.57–1.06)
730–1094 DDDs (2 years–3 years)	30	1419	1.16 (0.81–1.68)	0.95 (0.65–1.37)	0.88 (0.60–1.28)
1095 or more DDDs (3 or more years)	49	3258	1.00 (0.75–1.34)	0.83 (0.62–1.12)	0.75 (0.55–1.02)

^a^First adjustment controlled for date of dx, age, ethnic group, deprivation, urban/rural status, public/private status of the facility, register, stage, grade, mode of detection, lymphovascular invasion, and receptor status

^b^Second adjustment controlled for the previous covariates as well as other drug use and hospitalised comorbidities (other drugs including statins, NSAIDs and aspirin, ACEIs, ARBs, and diuretics. Comorbidities including any cardiac condition as yes/no, diabetes, stroke, COPD, and peripheral vascular disease). Other drug covariates were modelled in the same fashion as beta blockers (except for dose analysis, in which other drugs were classified as user/nonuser and modelled as time-varying covariates)

^c^DDDs refer to daily defined doses

^d^The p value for linear trend for the fully adjusted dose analysis was 0.1940

When comparing BB users to a BB nonuser group who used another antihypertensive (Table 4), a 24% increased risk of BCD was found (fully adjusted HR = 1.24; 1.05–1.47). This increased risk was reduced to a null effect after lagging medications by 2 years (HR = 0.98; 0.77–1.25).

**Table 4:** Associations of breast cancer-specific survival with post-diagnostic use of beta blockers (vs non-use) in breast cancer patients, using a comparison group of nonusers who used another antihypertensive

Medication usage after diagnosis	No. breast cancer deaths	No. person-years	Unadjusted HR (95% CI)	Adjusted^a^ HR (95% CI)	Fully adjusted^b^ HR (95% CI)
BB nonusers who used another antihypertensive	320	17,709	1.00	1.00	1.00
BB user	305	12,487	1.37 (1.18–1.61)	1.20 (1.02–1.41)	1.24 (1.05–1.47)
BB user, 6-month lag	252	10,946	1.34 (1.13–1.60)	1.19 (0.99–1.42)	1.21 (1.01–1.46)
BB user, 1-year lag	220	9496	1.38 (1.15–1.66)	1.25 (1.03–1.51)	1.29 (1.06–1.57)
BB user, 2-year lag	131	6965	1.06 (0.85–1.33)	0.96 (0.76–1.21)	0.98 (0.77–1.25)

^a^First adjustment controlled for date of dx, age, ethnic group, deprivation, urban/rural status, public/private status of the facility, register, stage, grade, mode of detection, lymphovascular invasion, and receptor status

^b^Second adjustment controlled for the previous covariates as well as other drug use and hospitalised comorbidities (other drugs including statins, NSAIDs and aspirin, ACEIs, ARBs, and diuretics. Comorbidities including any cardiac condition as yes/no, diabetes, stroke, COPD, and peripheral vascular disease). Other drug covariates were modelled in the same fashion as beta blockers

In the analysis considering recurrence as the outcome (Table 5), there was no statistically significant association found between BB use and BCR (HR = 1.06; 0.90–1.25). In the dose analysis, there was no longer an elevated risk associated with the initial 0–3 months of use (fully adjusted HR = 1.13; 0.87–1.49, Table 5). The risk was inconsistent across dose categories; however, there was a trend of a reduced risk with increasing duration of use among users (p for trend = 0.0061).

**Table 5:** Associations of breast cancer recurrence with post-diagnostic use of beta blockers (vs non-use) in breast cancer patients, by total dose

Medication usage after diagnosis	No. breast cancer recurrences	No. person-years	Unadjusted HR (95% CI)	Adjusted^a^ HR (95% CI)	Fully adjusted^b^ HR (95% CI)
BB nonuser	989	47,349	1.00	1.00	1.00
BB user	227	9619	1.12 (0.97–1.29)	1.02 (0.87–1.19)	1.06 (0.90–1.25)
1–90 DDDs (0–3 months)	60	2454	1.24 (0.96–1.62)	1.10 (0.85–1.44)	1.13 (0.87–1.49)
91–181 DDDs (3–6 months)	42	1391	1.36 (0.99–1.85)	1.29 (0.94–1.77)	1.33 (0.96–1.83)
182–272 DDDs (6–9 months)	25	950	1.13 (0.76–1.68)	1.06 (0.71–1.58)	1.10 (0.73–1.66)
273–364 DDDs (9 months–1 year)	19	777	1.08 (0.69–1.70)	0.96 (0.61–1.53)	1.00 (0.63–1.59)
365–729 DDDs (1 year–2 years)	52	1775	1.30 (0.98–1.72)	1.16 (0.87–1.55)	1.20 (0.90–1.62)
730–1094 DDDs (2 years–3 years)	12	928	0.61 (0.35–1.09)	0.54 (0.31–0.96)	0.56 (0.32–1.01)
1095 or more DDDs (3 or more years)	17	1343	0.67 (0.41–1.09)	0.62 (0.38–1.01)	0.65 (0.39–1.06)

^a^First adjustment controlled for date of dx, age, ethnic group, deprivation, urban/rural status, public/private status of the facility, register, stage, grade, mode of detection, lymphovascular invasion, and receptor status

^b^Second adjustment controlled for the previous covariates as well as other drug use and hospitalised comorbidities (other drugs including statins, NSAIDs and aspirin, ACEIs, ARBs, and diuretics. Comorbidities including any cardiac condition as yes/no, diabetes, stroke, COPD, and peripheral vascular disease). Other drug covariates were modelled in the same fashion as beta blockers (except for dose analysis, in which other drugs were classified as user/nonuser and modelled as binary time-varying covariates)

^c^Restricted to patients with stage 1, stage 2, or stage 3a cancers. Patients with an ‘unknown’ stage were excluded in this analysis

^d^DDDs refer to daily defined doses

^e^The *p* value for linear trend for the fully adjusted dose analysis was 0.0061

## Discussion

There was a small and nonstatistically significant increased risk between any BB use after breast cancer diagnosis and BCD in this large NZ population-based study of patients with breast cancer after adjustment for demographic and clinical factors, comorbidities, and other medication use. However, further analyses revealed a complex pattern: there was an increased risk associated with the initial few months of use, but a decreasing risk with longer-term use, with evidence of a dose–response trend indicative of a potential protective effect of long-term BB use on BCD.

Our primary finding is consistent with a number of previous studies indicating no significant association between BB use and BCD in their fully adjusted analyses [16, 20, 22–25], whilst a number have found BBs to be protective [16–18], and one to increase the risk of BCD [26]. For example, a study of 466 patients with breast cancer in England found hypertensive patients treated with BBs to have a 71% reduction in BCD (HR = 0.29; 0.12–0.71) [17]. Conversely, a study of 14,766 patients with breast cancer aged between 66 and 80 in the USA found BBs to increase the risk of BCD by 41% (HR = 1.41; 1.07–1.84) [26]. Another English study of 9817 patients with breast cancer found BB use to be associated with a smaller and not statistically significant increased risk of BCD (HR = 1.20; 0.92–1.57) [22]. All these studies considered BB use as a time-varying covariate. One other study indicated no association between BB use and BCD [21], and one found BBs to be protective [19]; however, these two studies did not account for the time-varying nature of medication use.

There are many reasons why results of studies examining medication use and cancer outcomes may vary. For example, studies which do not account for the time-varying nature of medication use are more likely to observe a protective effect through the introduction of immortal time bias [19, 21, 28, 40]. Furthermore, the definition of medication use varies from study to study, with some considering BB use prior to diagnosis, some after diagnosis, and some in other periods. In our study, we only considered BB use after diagnosis, arguably the most clinically relevant exposure period. Indications for BBs have also changed over time, and heart failure is now their most common indication, whereas BBs were commonly prescribed for hypertension 15–20 years ago [9, 46]. The general health of patients medicated with BBs has therefore likely changed from a healthier group to a less healthy group over time, with corresponding implications for their risk of BCD.

We found the risk of BCD to decrease with increasing doses of BBs (*p* for trend = 0.0005), with a HR of 1.40 (1.14–1.73) for the initial 0 to 3 months of use, and a HR of 0.55 (0.34–0.88) for 3 or more years of use. After the equivalent of a year’s use, the risk was decreased with increasing duration of use, which may be suggestive of a protective effective associated with long-term BB use. Previous literature investigating dose–response effects of BBs has been sparse; however, our results are in contrast to two previous papers (although neither paper reported a statistically significant dose effect) [20, 22], whilst one found no pattern [26], and one also found a similar pattern to ours for a non-selective BB, propranolol [16]. To our knowledge, ours is the first paper to study the long-term (i.e. 3 years +) effect of BBs on BCD and find a consistent dose–response pattern indicating a protective effect associated with long-term use. It is possible that this relationship can partly be explained by a healthy user effect [47], whereby those who tolerated BBs well and were healthier in general were more likely to stay on the medication for longer periods. However, the fact that this pattern was not reproduced for other cardiovascular medications (e.g. calcium channel blockers, Table 3) only serves to strengthen the suggestion that there may indeed be a causal and protective relationship between long-term BB use and BCD. It seems plausible that the increased risk of BCD we observed in the initial period of use (between 0 and 3 months) can predominantly be explained by women being medicated for symptomatic conditions towards the end of life. These women then go on to die soon after and are coded as dying from breast cancer. It might be considered that this end-of-life dispensing is creating a spurious dose–response effect for BBs; however, a statistically significant p for trend value was observed even after excluding these users from the analysis (Supplementary Table S2). The protective effect observed in long-term users can likely be explained by the absence of any ‘end of life’ prescribing effect, as well as an increasing systemic uptake of BBs over time, with higher dosings seemingly necessary to elicit any real protective effect.

The hypothesis that increased risk associated with the first few months of use is driven by short-term use towards the end of life is supported by an amalgamation of evidence. Firstly, there was a clear pattern in women’s median time to death/last follow-up from their first BB dispensing by dose category: those who took BBs in lower doses consistently had a shorter time interval between their first dispensing and death/last follow-up than those who took BBs for longer (Supplementary Table S3). Secondly, there was no longer a statistically significant increased risk in short-term users when considering BCR as the outcome (Table 5). This supports the idea that a significant number of women are dispensed a BB after a recurrence (perhaps because of complications associated with the recurrence) and then go on to die soon after. Lastly, the short-term use category was the dose category with the highest proportion of ‘new’ users (new users meaning those who did not have a BB dispensing in the year prior to diagnosis) relative to any other dose category (Supplementary Table S4). This is consistent with their first dispensing being towards the end of life, rather than continuing use initiated prior to their breast cancer diagnosis. Furthermore, the short-term user category had the highest proportion of breast cancer deaths relative to any other dose category.

It has been suggested that breast cancer and CVD deaths are sometimes misclassified, with one study suggesting that women’s cancer diagnosis is often perceived as the overriding medical priority in patients with both cancer and CVD [48]. However, we found similar associations between BBs and BCD and BBs and BCR, which argues against misclassification of cause of death as a major issue in our cohort (as most women who die from breast cancer have had a BCR [49]). Furthermore, evidence from an Australian study assessing the accuracy of national cause of death coding found the specificity of cancer deaths to be 99.2% [50], showing that very few deaths were wrongly attributed to cancer.

When BB users were compared with a more similar comparison group (BB nonusers who used another antihypertensive), we observed a 24% increased risk of BCD after adjustment for confounding variables. This result may be explained by BB dispensings towards the end of life, as the HR decreased from 1.24 (1.05–1.47) to 0.98 (0.77–1.25) when lagging medication times by 2 years. Therefore, it appears that any differences in the HRs between different comparison groups can mostly be attributed to differences in end-of-life care between the two groups, rather than any fundamental differences in their underlying characteristics.

Finally, adjusting for the competing risk of death from other causes or analysing early-stage patients only did not substantially affect the HR. Moreover, when we applied these types of analyses to our other results, the HR did not change to a degree that would alter the interpretation of our results.

The primary strength of our study is that we had a large cohort of patients with breast cancer followed up over a relatively long time period sourced from four population-based databases. The Auckland and Waikato databases have been checked against the National Cancer Registry and found to be at least 99% complete, and the registry data we used contains more comprehensive and accurate information than the national data sources [51–53]. Our pharmaceutical data were derived from a high quality and automated national database, and there was no recall bias [54] associated with medication records as a result. Furthermore, unlike many other countries, New Zealand records medication dispensings instead of prescriptions, which are a stronger proxy for medication adherence. We also conceptualised medication use as time-varying covariates, and therefore avoided the introduction of immortal time bias that invariably biases results in favour of the medication [40].

Our study also has limitations. We did not have access to primary care data, which meant that our comorbidity data were restricted to hospital admissions in the relevant timeframe. Furthermore, this limited access to a range of potential confounders such as body mass index, alcohol intake, and smoking status, all of which would generally be available through general practitioner records. However, these limitations in residual confounders were somewhat mitigated by the use of a more balanced comparison group. The most commonly prescribed BB in New Zealand is a selective BB, metoprolol [9], which was used by 78% of the BB users in our study. Therefore, we did not have the power to explore the relationship between non-selective BBs (such as propranolol and carvedilol) and BCD. Several preclinical studies have suggested that non-selective BBs may have a higher efficacy in inhibiting pathways involved in breast cancer progression and metastasis [15, 55]. Finally, we did not adjust for breast cancer treatment such as chemotherapy and radiotherapy. However, it is likely that any treatment variables would have been highly correlated with other variables in our model such as stage and grade.

In conclusion, there was only a weak and non-significant association between post-diagnostic BB use and BCD in this large population-based study of NZ patients with breast cancer. Further analyses showed an increased risk associated with the first few months of use, likely to be due to use towards the end of life. For longer-term use, risk of death decreased with increasing duration of use, suggesting that long-term BB use may confer a protective effect on BCD. Further research is warranted to assess if this relationship is replicated in other clinical settings.

## Supplementary Information

Below is the link to the electronic supplementary material.

Supplementary file1 (DOC 59 kb)

## Data Availability

The datasets used in this study contain personal information and are not publicly available, but may be requested from the Breast Cancer Foundation New Zealand and the Ministry of Health (NZ).
