# Supplementary material for: Post-diagnostic beta blocker use and breast cancer-specific mortality: a population-based cohort study
Source: Breast Cancer Res Treat. 2022 Mar 14;193(1):225–35. doi: 10.1007/s10549-022-06528-0 (PMC8993732; doi:10.1007/s10549-022-06528-0)
Supplement: Supplementary file 1 — Supplementary file1 (DOC 59 kb) [file 10549_2022_6528_MOESM1_ESM.doc]

**Supplementary tables**

**Supplementary Table 1. Associations of breast cancer specific survival with postdiagnosis use of beta blockers in breast cancer patients.**

| **Medication Usage After Diagnosis** | **No. Breast cancer deaths** | **No. person-years** | **Unadjusted HR (95% CI)** | **Adjusted^a^ HR (95% CI)** | **Adjusted^b^ HR (95% CI)** | **Adjusted^c^ HR (95% CI)** | **Fully adjusted^d^ HR (95% CI)** |
| --- | --- | --- | --- | --- | --- | --- | --- |
| BB nonuser | 1,036 | 60,301 | 1.00 | 1.00 | 1.00 | 1.00 | 1.00 |
| BB user | 305 | 12,487 | 1.49 (1.31-1.70) | 1.14 (1.00-1.31) | 1.16 (1.01-1.33) | 1.15 (1.00-1.33) | 1.11 (0.95-1.29) |

*^a^First adjustment controlled for* *date of dx, age, ethnic group, deprivation, urban/rural status, public/private status of the facility, and register.*

*^b^Second adjustment controlled for the previous covariates as well as* *stage, grade, mode of detection, lymphovascular invasion, and receptor status.*

*^c^Third adjustment controlled for the previous covariates as well hospitalised comorbidities. Comorbidities included any cardiac condition as yes/no, diabetes, stroke, COPD, and peripheral vascular disease.*

*^d^Fourth adjustment controlled for the previous covariates as well as other drug use including statins, NSAIDs and aspirin, ACEIs, ARBs, and diuretics. Other drug covariates were modelled in the same fashion as beta blockers.*

**Supplementary Table 2. Associations of breast cancer specific survival with postdiagnosis use of beta blockers in breast cancer patients, by total dose (excluding the initial 0-3 months of use).**

| **Medication Usage After Diagnosis** | **No. Breast cancer deaths** | **No. person-years** | **Unadjusted HR (95% CI)** | **Adjusted**^a^ **HR (95% CI)** | **Fully adjusted**^b^ **HR (95% CI)** |
| --- | --- | --- | --- | --- | --- |
| BB nonuser | 1,036 | 60,301 | 1.00 | 1.00 | 1.00 |
| 91-181 DDDs (3-6 months) | 45 | 1,782 | 1.43 (1.06-1.93) | 1.22 (0.90-1.66) | 1.13 (0.83-1.55) |
| 182-272 DDDs (6-9 months) | 31 | 1,225 | 1.42 (0.99-2.03) | 1.26 (0.87-1.81) | 1.23 (0.85-1.78) |
| 273-364 DDDs (9 months-1 year) | 27 | 1,001 | 1.50 (1.02-2.20) | 1.19 (0.80-1.75) | 1.13 (0.76-1.68) |
| 365-729 DDDs (1 year-2 years) | 53 | 2,325 | 1.34 (1.01-1.76) | 1.01 (0.76-1.35) | 0.96 (0.71-1.28) |
| 730-1094 DDDs (2 years-3 years) | 21 | 1,180 | 1.12 (0.73-1.74) | 0.78 (0.50-1.21) | 0.78 (0.50-1.23) |
| 1095 or more DDDs (3 or more years) | 19 | 1,774 | 0.81 (0.51-1.28) | 0.55 (0.34-0.88) | 0.55 (0.34-0.88) |

*^a^First adjustment controlled for date of dx, age, ethnic group, deprivation, urban/rural status, public/private status of the facility, register, stage, grade, mode of detection, lymphovascular invasion, and receptor status.*

*^b^Second adjustment controlled for the previous covariates as well as other drug use and hospitalised comorbidities (other drugs including statins, NSAIDs and aspirin, ACEIs, ARBs, and diuretics. Comorbidities including any cardiac condition as yes/no, diabetes, stroke, COPD, and peripheral vascular disease). Other drug covariates were classified as user/nonuser and modelled as time varying covariates.*

*^c^DDDs refer to daily defined doses.*

*^d^The p for trend value for the fully adjusted dose analysis was 0.0034.*

Supplementary Table 3. Median time to death/last follow up from women’s first BB dispensing, by dose category.

| Dose category | Number of users who died due to breast cancer | Median time to death (in years) from their first BB dispensing | Number of users who died due to any cause | Median time to death (in years) from their first BB dispensing | Number of users who didn’t die | Median time to last follow up (in years) from their first BB dispensing |
| --- | --- | --- | --- | --- | --- | --- |
| 1-90 DDDs (0-3 months) | 109 | 0.72 | 197 | 0.61 | 553 | 1.73 |
| 91-181 DDDs (3-6 months) | 45 | 1.58 | 95 | 1.61 | 229 | 2.12 |
| 182-272 DDDs (6-9 months) | 31 | 1.80 | 64 | 2.06 | 245 | 2.35 |
| 273-364 DDDs (9 months-1 year) | 27 | 2.44 | 50 | 2.47 | 197 | 2.78 |
| 365-729 DDDs (1 year-2 years) | 53 | 2.68 | 130 | 3.08 | 482 | 3.78 |
| 730-1094 DDDs (2 years-3 years) | 21 | 3.77 | 64 | 3.69 | 262 | 5.11 |
| 1095 or more DDDs (3 or more years) | 19 | 5.25 | 87 | 5.78 | 540 | 6.74 |

**Supplementary Table 4. Proportion of ‘new’ users and proportion of users who died due to breast cancer, by dose category.**

| Dose category | Percentage of ‘new’ users^a^ | Percentage of users who died due to breast cancer |
| --- | --- | --- |
| 1-90 DDDs (0-3 months) | 77.47 | 14.53 |
| 91-181 DDDs (3-6 months) | 49.38 | 13.89 |
| 182-272 DDDs (6-9 months) | 38.51 | 10.03 |
| 273-364 DDDs (9 months-1 year) | 34.82 | 10.93 |
| 365-729 DDDs (1 year-2 years) | 25.65 | 8.66 |
| 730-1094 DDDs (2 years-3 years) | 21.78 | 6.44 |
| 1095 or more DDDs (3 or more years) | 11.96 | 3.03 |

*^a^A ‘new’ user was defined as women who did not have a BB dispensing in the year prior to their breast cancer diagnosis.*
